# Tailoring the Size and Shape—New Path for Ammonium Metavanadate Synthesis

**DOI:** 10.3390/ma12203446

**Published:** 2019-10-21

**Authors:** Marta Prześniak-Welenc, Małgorzata Nadolska, Barbara Kościelska, Kamila Sadowska

**Affiliations:** Faculty of Applied Physics and Mathematics, Gdansk University of Technology, Narutowicza 11/12, 80-233 Gdansk, Poland

**Keywords:** ammonium vanadates, hydrothermal synthesis, morphology control

## Abstract

Ammonium metavanadate, NH_4_VO_3_, plays an important role in the preparation of vanadium oxides and other ammonium compounds, such as NH_4_V_3_O_8_, (NH_4_)_2_V_3_O_8_, and NH_4_V_4_O_10_, which were found to possess interesting electrochemical properties. In this work, a new route for the synthesis of NH_4_VO_3_ is proposed by mixing an organic ammonium salt and V_2_O_5_ in a suitable solvent. The one-step procedure is carried out at room temperature. Additionally, the need for pH control and use of oxidants necessary in known methods is eliminated. The mechanism of the NH_4_VO_3_ formation is explained. It is presented that it is possible to tailor the morphology and size of the obtained NH_4_VO_3_ crystals, depending on the combination of reagents. Nano- and microcrystals of NH_4_VO_3_ are obtained and used as precursors in the hydrothermal synthesis of higher ammonium vanadates. It is proven that the size of the precursor particles can significantly affect the physical and chemical properties of the resulting products.

## 1. Introduction

Ammonium metavanadate (NH_4_VO_3_) plays an important role in the preparation and purification of vanadium compounds, including, for example, NH_4_V_3_O_8_, NH_4_V_4_O_10,_ (NH_4_)_0.6_V_2_O_5_, and V_2_O_5,_ V_6_O_13_ [1,2,3,4,5,6,7]. These vanadium oxides and their derivatives are of huge interest, mainly due to their Li-ion intercalation properties. Therefore they are considered as a favorable cathode material inthe secondary Li-ion batteries (LIBs) [1,4,7]. Recent studies have shown that vanadium oxide derivatives can also be successfully used in multivalent ion batteries, such as calcium-ion [8], magnesium-ion [9], and zinc-ion [10,11,12] batteries. Moreover NH_4_VO_3_ is used as an efficient and mild catalyst for the synthesis of α-hydroxyphosphonate derivatives, which act as inhibitors of a diverse group of enzymes e.g. HIV protease [13]. Ammonium metavanadate can also be used as an electrolyte additive in the electrochemical anodization of magnesium alloy surface to improve its corrosion resistance [14]. However, one of the main drawbacks connected with the commercial usage of ammonium metavanadate in the mentioned applications is the cost of the synthesis of this precursor. The industrial production of NH_4_VO_3_ is usually a multi-step procedure, carried out under harsh conditions (oxidizing agents, alkalines) and elevated temperature [15,16]. Moreover, ammonium chloride or other inorganic salts, serving as an ammonium ions source, need to be used in the substantial excess to enable the efficient precipitation of the desired product. Therefore, next to the high cost of the production, the obtained NH_4_VO_3_ suffers from poor particle size distribution and possibly high concentration of impurities. The morphology of the precursor strongly affects the properties of the final product, especially the electrochemical performance, which is of the priority, when referring to LIBs. Therefore, novel methods of ammonium metavanadate synthesis are sought, with particular interest to approaches enabling NH_4_VO_3_ crystals size and shape control. Controllable synthesis of ammonium metavanadate is of importance not only due to the potential electrochemical applications. Here, it should be noted that several examples of NH_4_VO_3_ use as catalyst can be found in the literature [17,18,19]. It is known that the size of the catalyst particles strongly influences its catalytic performance [20,21]. Therefore, simple and efficient synthesis of uniform and nano-sized NH_4_VO_3_ particles would pave the way for their wider application spectrum. 

In this paper, we report a simple, low temperature, one-pot approach for ammonium metavanadate synthesis, while using V_2_O_5_ and chosen organic ammonium salt as substrates and suitable solvent. Beneficially, the size and shape of the obtained NH_4_VO_3_ crystals can be simply controlled by choosing type of the solvent and type of the organic ammonium salt. Therefore, the properties of the synthesized material can be tailored for a specific purpose.

## 2. Materials and Methods 

### 2.1. Materials

Formamide (99.5%, ACROS Organics, New Jersey, NJ, USA), ammonium formate (99%, ACROS Organics, New Jersey, NJ, USA), ammonium acetate (97%, Alfa Aesar, Kandel, Germany), V_2_O_5_ (99.2%, Alfa Aesar, Kandel, Germany), NH_4_VO_3_ (99%, Sigma Aldrich, Saint Louis, MO, USA), and oxalic acid dihydrate (C_2_H_2_O_4_·2H_2_O, L.P P-H “OH”) were used without further purification. MiliQ water (0.05 µS cm^−1^) was used in all experiments. 

The general procedure was as follows. To 50 mL of 1.25 mol/L solution of ammonium organic salt (ammonium formate or ammonium acetate) in appropriate solvent (water or formamide) 50 mg of V_2_O_5_ was added. The mixture was agitated for several minutes at RT and then left for 12 h. From the initially yellow solution, white solids precipitated, which were separated by centrifugation (2 min, 14000 rpm). After washing several times with ethanol, the white crystalline product was dried overnight at RT under reduced pressure (0.01 bar). The obtained samples were denoted, as presented in Table 1.

#### Hydrothermal Synthesis of Ammonium Vanadates

In a typical procedure, 0.2 g NH_4_VO_3_ (commercial or AF/F) and 0.2 g C_2_H_2_O_4_·2H_2_O were dissolved in 7 mL of deionised water under magnetic stirring (300 rpm). Subsequently, the obtained solutions were placed into a stainless-steel autoclave (Series 4680, Parr Instrument Co., Moline, IL, USA) with a capacity of 1800 mL and then kept for 72 h at 180 °C. After that time, the autoclave was evacuated with a rate of ca. 1 bar/min and then cooled to room temperature naturally. Finally, the obtained precipitates were washed several times with deionised water and then dried at 40 °C under vacuum.

### 2.2. Methods

The phase composition of obtained samples was examined by X-ray diffraction method (XRD) by Philips X’Pert diffractometer system (Royston, UK) while using CuKα radiation in range of 5–80° of 2Θ. The FT-IR spectra were recorded at room temperature while using a Perkin-Elmer spectrometer (model Frontier FTIR MIR/FIR) (Waltham, MA, USA). The FT-IR spectra of the samples that were pressed into KBr pellets with constant measure material concentration (0.5%) were collected in the wave number range 4000–400 cm^−1^ (mid IR region) while using the KBr beam splitter. The surface morphologies of the samples were studied by a FEI Company Quanta FEG 250 scanning electron microscope (SEM) (Waltham, MA, USA), mounting the analyzed sample on a carbon conductive tape The thermogravimetric analysis (TGA) was performed under argon atmosphere (flow rate 60 cm^3^ min^−1^) with heating rate 10 °C/min. from 40 °C to 550 °C while using Netzsch STA 449 F1 Jupiter® (Netzsch, Selb, Germany). Constant sample mass (6 ± 0.5 mg) was used. The thermal behavior has also been studied with mass spectrum (MS). The gases that come out from sample during heating were monitored by the quadruple mass spectrometer Netzsch QMS 403 Aëolos (Netzsch, Selb, Germany).Raman spectra were recorded while using Renishaw InVia spectroscope (Renishaw, UK) with argon ion laser operating at 514.5 nm focused through a 50× objective. Collected light was dispersed through a triple monochromator and detected with a charge-coupled device. The spectra were collected in the dark, with a resolution of 2 cm^−1^ in the range of 100–3000 cm^−1^.

## 3. Results and Discussion

### 3.1. Structural Analysis

Ammonium metavanadate was obtained in a simple, low cost, and one-step method. In the first experiment, ammonium formate was used as a source of ammonium ion and formamide as a solvent. According to the literature, formamide is as an effective delaminating agent that is used in the liquid-phase exfoliation of V_2_O_5_ [22]. Therefore, it was considered to be a solvent of choice. Ammonium formate possessing the same structural core as a formamide was an apparent selection. Analogously, the reaction was carried out while using water as a solvent, keeping the same ratio of reagents. Water was used as a cheaper and environmentally friendly solvent. It was found out that the reaction proceeded in both solvents. However, it was visible by the naked eye, whereby the morphology of the obtained products differs dramatically. Fine, white powder was collected from the reaction that was carried out in the formamide, whereas bulky, uniform crystals were obtained in the aqueous environment. 

**Figure 1:**
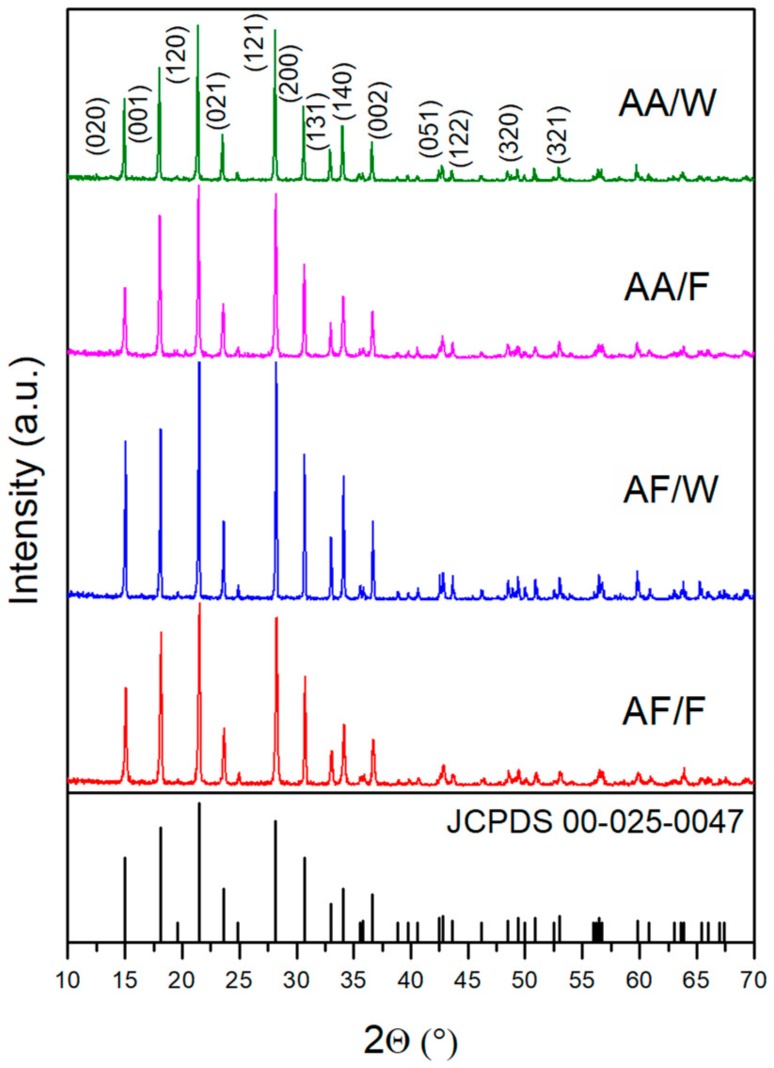
X-ray diffraction method (XRD) patterns of samples AF/F, AF/W, AA/F, and AA/W.

X-ray diffraction patterns were recorded to confirm the phase purity and crystallinity of the obtained samples. Figure 1 illustrates the part of XRD patterns (10–70° of 2θ) of AF/F, AF/W, AA/F, and AA/W. The observed diffractions peak for all samples match very well with JCPDS Card no. 00-025-0047, which corresponds to the NH_4_VO_3_ orthorhombic structure with a space group of Pmab (No. 57) with lattice parameter values of a = 5.827 Å, b = 11.782 Å, and c = 4.905 Å. Moreover, no signals of other phases were detected, which indicated the high purity of obtained NH_4_VO_3_ micro- and nanocrystals.

**Figure 2:**
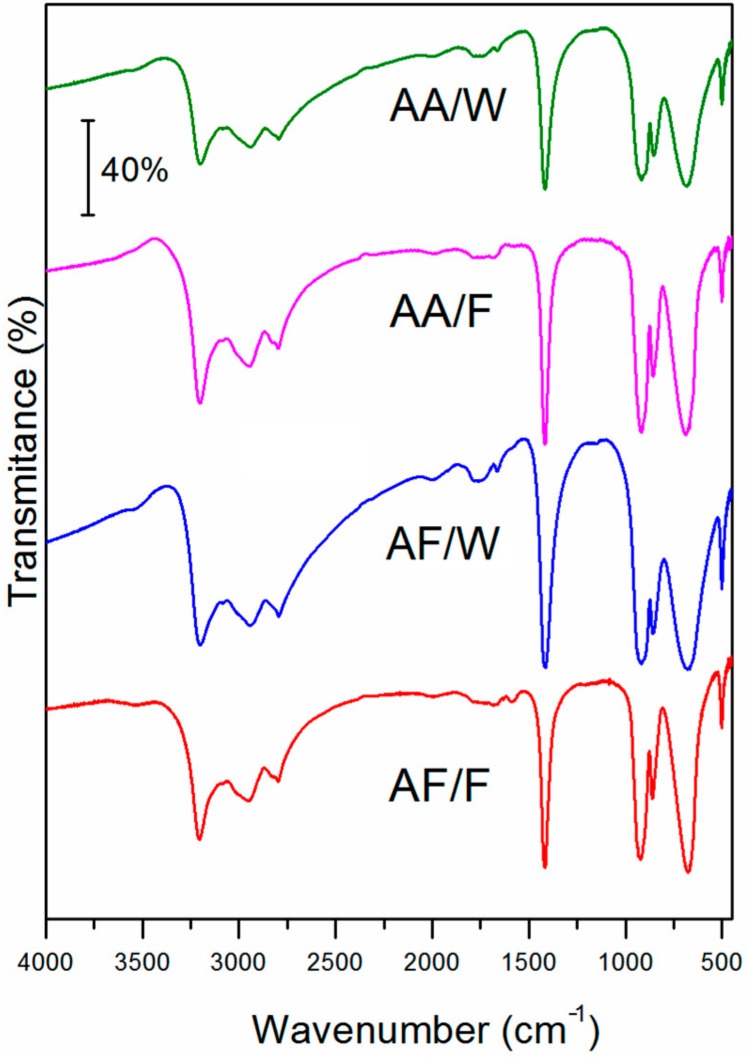
FTIR spectra of samples AF/F, AF/W, AA/F, and AA/W.

FTIR spectroscopy was used to further analyze the obtained samples. Figure 2 presents the FTIR spectra of four analyzed samples. The spectra recorded for all samples are identical and they are in agreement with the reference spectrum of ammonium metavanadate (NIST Chemistry WebBook, SRD 69), which confirms the identity of the synthesized products (see Supplementary Materials, Figure S1). The bands centered at 3200, 2945, and 2796cm^−1^ are assigned to the stretching vibration of bonds in the NH_4_^+^. Next, the characteristic band for NH_4_^+^, located at 1414 cm^−1^, is due to N-H in plane vibration mode. The strong band at 919 cm^−1^ refers to V=O stretching and other bands that are visible in the region between 860 cm^−1^ and 500 cm^−1^ are attributed to V-O-V bonds vibrations [23,24].

**Figure 3:**
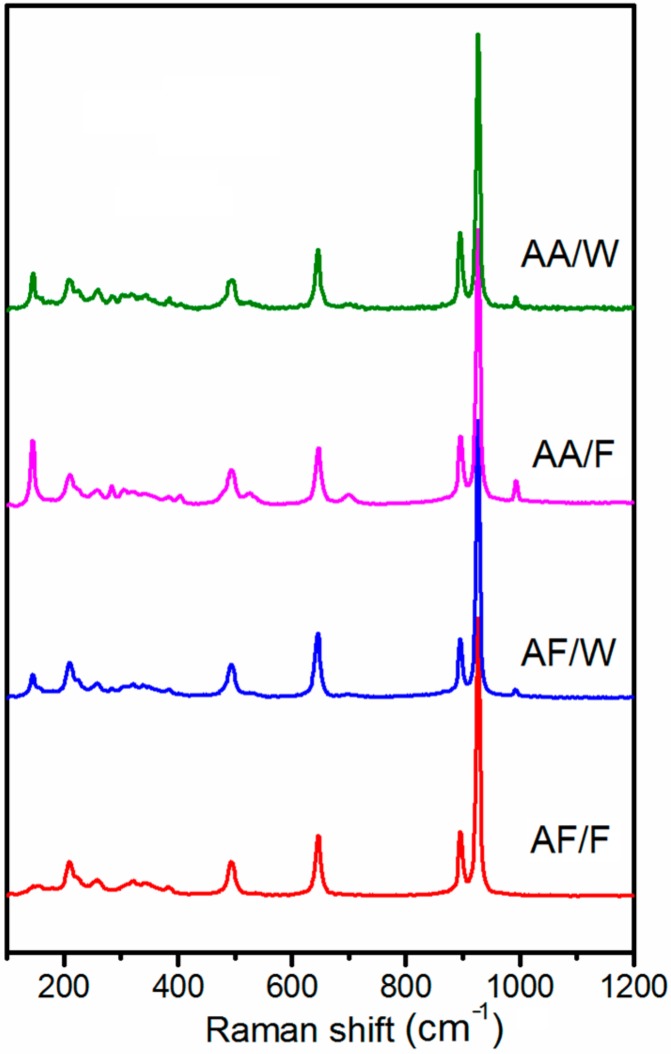
Raman spectra of samples AF/F, AF/W, AA/F, and AA/W.

Being complementary to FTIR, Raman spectroscopy was also used to confirm the structure of the samples. All spectra, as presented in Figure 3, conform to the NH_4_VO_3_ spectrum (see Figure S2). The main peak, positioned at 926 cm^−1^ corresponding to VO_2_ symmetrical vibrations, is followed by a smaller band at 895 cm^−1^ that arises from asymmetrical VO_2_ vibrations. The other visible bands at 647 and 495 cm^−1^ are connected with V-O-V asymmetric and symmetric stretching, respectively. At lower frequency region (400–200 cm^−1^), several bands of low intensity can be observed, and they refer to VO_2_ bending and NH_4_^+^ stretching [24]. Below 200 cm^−1^, the Raman bands are assigned to the lattice modes and they are generally observed for layered structures. The different intensities may be due to the different morphologies of the analyzed samples.

**Figure 4:**
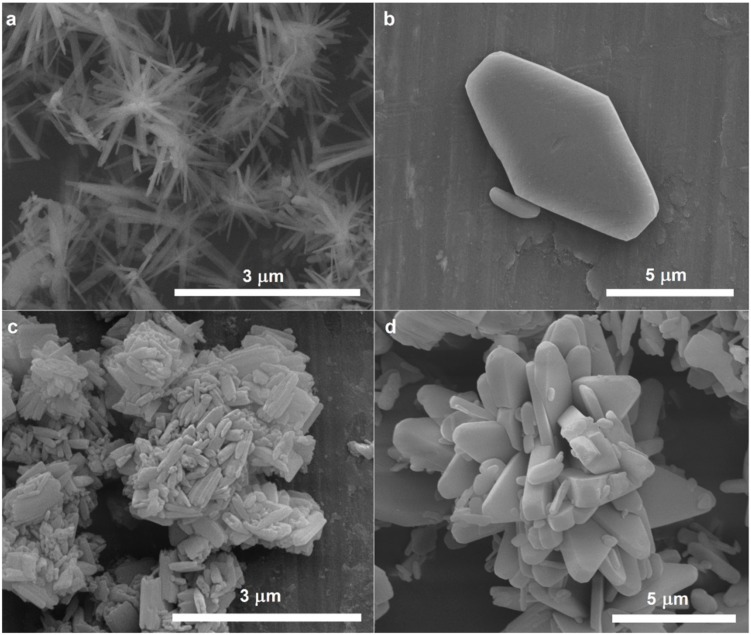
Scanning electron microscope (SEM) images of (**a**) AF/F; (**b**) AF/W; (**c**) AA/F and (**d**) AA/W.

The SEM analysis further revealed morphological differences. As can be seen in Figure 4a, the AF/F sample adopted flower-like structure, with dimensions between 1.5 µm and 3 µm. The length of single crystals forming a flower-like structure was in the range of 0.5–1 µm, their width was c.a. 0.2–0.5 µm, and their thickness was in the nanoscale and equal to 20–80 nm. Much bigger crystals can be seen in Figure 4b, presenting the morphology of AF/W sample. The obtained crystals were casketoids in shape, with a length of 7–10 µm, width 4.5–5.5 µm, and thickness 0.5–1.5 µm. 

The same protocol was performed in both solvents (that is formamide and water), using, however, ammonium acetate instead of ammonium formate in order to verify whether the procedure can be broadened to other salts. Again, both of the approaches succeeded in NH_4_VO_3_ preparation (see XRD results, Figure 1), and again they lead to different morphologies. When the reaction was carried out in formamide, using ammonium acetate as an ammonium cations source (AA/F sample), conglomerated crystals of elongated shape were prepared, as can be seen in Figure 4c. Their average length was between 0.3 µm and 0.7 µm, width: 0.1–0.4 µm, and thickness: 0.1–0.2 µm. In the case of the reaction that was conducted in water, the casketoidal shape of crystals was also observed, however the crystals were agglomerated (Figure 4d). They were 3–10 µm long, 1.5–2 µm wide, and 0.4–0.8 µm thick. 

**Figure 5:**
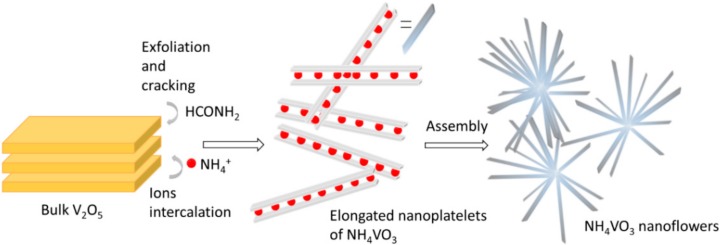
Scheme of proposed mechanism of NH_4_VO_3_nanoflowers formation.

The SEM images clearly show that the morphology of the end product can be nicely tuned by changing the type of ammonium salt and the solvent. It can be concluded that the used solvent affected crystal shapes and their size was more salt-type dependent. Moreover, the use of ammonium acetate resulted in more agglomerated samples. Additionally, mixtures of the solvents (water and formamide) and mixture of the salts (ammonium acetate and formate) were used, respectively (Supplementary Materials, Figures S3–S6). The SEM images (Figure 4a,c, Supplementary Materials, Figures S3d–f and S5) demonstrated that formamide, regardless of the salt type used, caused the delamination of starting oxide and enhanced the formation of elongated structures. Formamide was previously reported to be an effective solvent for V_2_O_5_ liquid exfoliation. Analyzing the SEM images, it can be stated that the intercalation of formamide molecules into V_2_O_5_ is the first step in the proposed synthesis. In Figure S3d, it can be clearly seen that the monolith of V_2_O_5_ flakes off, which results in the detachment of thin elongated shavings. Simultaneously, ammonium cations are dragged into the vanadium-oxide layers by means of the formate anions, forming ammonium metavanadate nanorods. Figure 5 presents the scheme of the process. 

The observation of initial solutions that were prepared in formamide and water supports the mechanism described above. If the precursor V_2_O_5_ was added into the solution of organic salt in formamide, the yellow slurry was formed, which, in time, changed color to white. The yellow color due to V_2_O_5_ disappeared, as solid vanadium oxide underwent delamination and ammonium ion intercalation to produce white ammonium metavanadates crystals. In contrast, when the V_2_O_5_ is added to the mixture of organic salt and water, the normally water-insoluble V_2_O_5_ creates a clear yellow solution. In aqueous solution, dioxovandium(V) ions VO_2_^+^ are formed, which undergo hydrolysis and dissociation, as follows, Equations (1) and (2):

VO_2_^+^ + H_2_O → VO(OH)_3_aq + H^+^ (1)

VO(OH)_3_aq → VO_2_(OH)_2_^−^+ H^+^ (2)

Protonation of VO_2_^+^ is a limiting step of the reaction and it requires the presence of strong acids. This restriction is negligible if complexing ligands are involved. If carboxylates are present in the solution, the RCOO^−^ might replace the OH^−^ to produce soluble complexes of structure VO(RCOO)_3_ andVO_2_(RCOO)_2_^−^, where R is CH_3_- or H- in our case. The formation of surface complexes, followed by the dissolution of acetate- or formate-vanadium(V) species, makes the aqueous solution homogenous. A similar observation was reported for vanadium-oxalate complexes [25]. The next step is the precipitation of the ammonium metavanadate from the reaction mixture, which results in bigger crystals of different shapes, as compared to the delamination product. 

### 3.2. Hydrothermal Synthesis of Higher Ammonium Vanadates from NH_4_VO_3_

As discussed before, the morphology and structure of the materials have great influence on their properties, which determine their potential usage. Ammonium metavanadate is a most frequently used precursor in the hydrothermal synthesis of other vanadates [26,27,28]. Therefore, hydrothermal synthesis was carried out in order to investigate an effect of the precursor morphology on the final product, while using commercial NH_4_VO_3_ and sample AF/F. Figure S7 presents the SEM image of commercial NH_4_VO_3_. Two aqueous solutions were prepared, keeping the same concentration of ammonium metavanadate (commercial or synthesized) and oxalic acid. Stirring the reaction mixtures before inserting them into autoclave revealed the first differences. The solution containing commercial NH_4_VO_3_ darkened to reddish-orange after one hour of stirring, whereas the solution of AF/F was pale yellow. After 72 h of hydrothermal reaction under 180 °C, the solids were separated and analyzed. The sample that was obtained from commercial NH_4_VO_3_ was denoted as HydroSigma and the latter one was denoted as HydroAF/F. 

**Figure 6:**
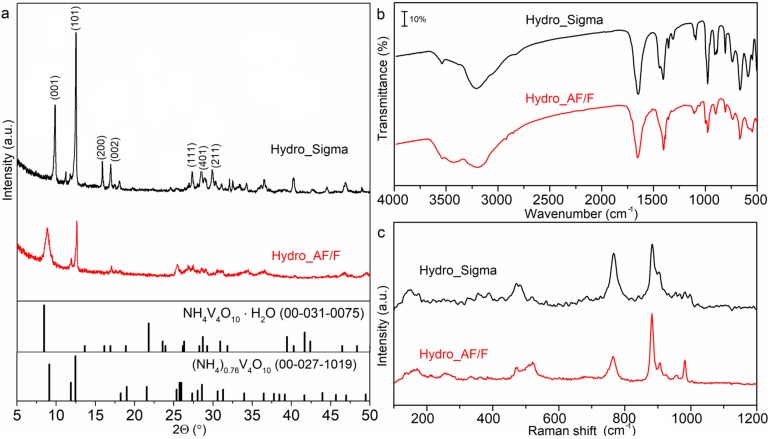
Structural analysis results: (**a**) XRD patterns of samples HydroSigma and HydroAF/F; (**b**) FTIR spectra of samples HydroSigma and HydroAF/F and (**c**) Raman spectra of samples HydroSigma and HydroAF/F.

The XRD results disclosed that synthesized material is a mixture of (NH_4_)V_4_O_10_·H_2_O (JCPDS no. 00-031-0075) and (NH_4_)_0.76_V_4_O_10_ (JCPDS no. 00-027-1019). As displayed in Figure 6a, diffractograms match well with the references, with the exclusion of the first peak of HydroSigma. Shifting of the first peak is well-known in the case of hydrated ammonium vanadates and is attributed to the water intercalation [12]. 

**Figure 7:**
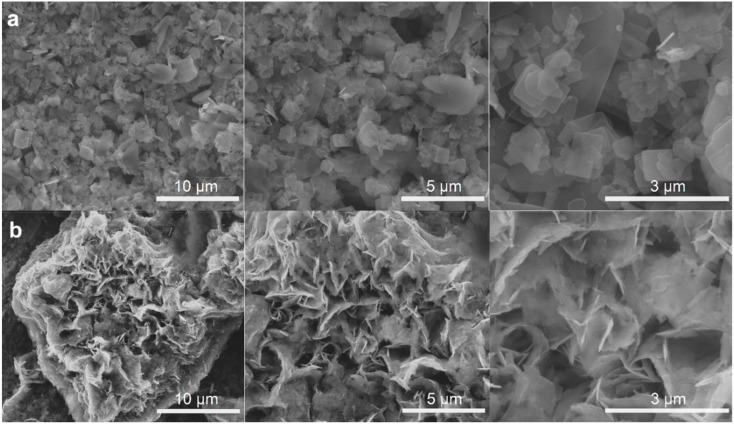
SEM images of (**a**) HydroSigma sample and (**b**) HydroAF/F sample with different magnifications.

By comparing the XRD patterns for both samples, it can be seen that the diffraction peaks of HydroAF/F are wider and less intense, which is typical for nanomaterials. Moreover, the average crystallite size of samples was calculated from the first diffraction peak while using the Scherrer equation, with the Sherrer constant of 0.9. As expected, the calculated average crystallite sizes are much smaller for HydroAF/F (14 nm) than for HydroSigma (150 nm), which is in accordance with SEM results (Figure 7). Analogously to the previous studies, FTIR and Raman spectroscopy measurements were carried out. In Figure 6b, the FTIR spectra of both samples are presented. Broad band centered at 3200 cm^−1^ and sharp band at 1412 cm^−1^, referring to NH_4_^+^ vibrations are observed for both samples. Characteristic for vanadium compounds bands are visible in the range of 1000–600 cm^−1^. The presence of crystalline water is revealed as a small band at 3540 cm^−1^ and strong band at 1645 cm^−1^. When comparing the intensities of V=O stretching band at ~1000 cm^−1^ with band of H_2_O stretching at 1645 cm^−1^ in two analyzed samples, it can be concluded that HydroAF/F contains more water. It is in agreement with XRD results, which showed that the percentage contribution of (NH_4_)V_4_O_10_·H_2_O was higher in the HydroAF/F sample, as compared to HydroSigma. Raman spectra revealed further differences. Figure 6c depicts the normalized Raman spectra. The main bands in HydroAF/F and HydroSigma spectra are positioned at 990, 880, 760, and 520 cm^−1^ and they correspond to VO_2_ symmetrical and asymmetrical vibrations and V-O-V asymmetric and symmetric stretching. Several bands of low intensity can be seen in the range of 150–500 cm^−1^, referring to VO_2_ bending and NH_4_^+^ stretching. The HydroSigma spectrum is more affected by the presence of non-stoichiometric phase, as the overlapping or quenching of some bands is observed. Moreover, the signals intensities were much lower in the case of Hydro Sigma sample. Neither, (NH_4_)V_4_O_10_·H_2_O nor (NH_4_)_0.76_V_4_O_10_ Raman spectrum has been reported so far. 

Figure 7 presents the SEM images of the manufactured materials. The morphological differences can be clearly seen. The sample HydroSigma is composed of uneven, differently shaped tiles with rounded edges. In the case of HydroAF/F thin, jagged flakes were created, assembling into porous structure. The time and temperature of the reaction were chosen based on the previously published papers. The reaction that was conducted within 72 h in 180 °C was found to be the most frequently used in the literature [26,27,28,29]. However, the influence of the precursor morphology on the final product has not been thoroughly studied. Our results proved that this factor cannot be neglected.

In summary, there are many methods for producing ammonium vanadium compounds; however, regardless of the technique, numerous synthesis parameters must be considered. In the production of nanomaterials, the precursor morphology is important, as it strongly affects the type and the properties of the final product.

## 4. Conclusions

A novel method of ammonium metavanadate synthesis was presented. The NH_4_VO_3_ was successfully obtained from V_2_O_5_, while using organic ammonium salt and formamide or water as a solvent. Diffractometry, Raman, and FTIR spectroscopy and SEM imaging analyzed all of the materials. Depending on the reaction parameters, differently sized and shaped structures were obtained. Micro- and nanocrystals were manufactured. Micro- and nanocrystalline NH_4_VO_3_ served as a precursor in hydrothermal reaction for higher ammonium vanadates synthesis. A mixture of (NH_4_)V_4_O_10_·H_2_O and (NH_4_)_0.76_V_4_O_10_ was obtained in both cases, however the contribution of each phase was different for the samples that were obtained from different precursor. The morphology was also strongly affected by the type of the precursor used. 

## Figures and Tables

**Table 1:** Nomenclature of obtained samples.

Solvent	Salt	
	Ammonium Formate	Ammonium Acetate
Water	AF/W	AA/W
Formamide	AF/F	AA/F

## Supplementary Materials

(FTIR, Raman, SEM of NH_4_VO_3_) is available in the online version of this article at https://www.mdpi.com/1996-1944/12/20/3446/s1, Figure S1: FTIR spectrum of ammonium metavanadate, Figure S2: Raman spectrum of ammonium metavanadate, Figure S3: SEM images presenting the structure of V_2_O_5_ used as a precursor (a and b) and intermediates formed in the reaction of V_2_O_5_ with ammonium formate in formamide (c and d), leading to flower-like nanostructural crystals of NH_4_VO_3_ (e and f), Figure S4: SEM images presenting structures of NH_4_VO_3_ obtained from V_2_O_5_ in water using ammonium formate (a and b), ammonium acetate (c and d), and equimolar mixture of ammonium formate and ammonium acetate (e and f), Figure S5: SEM images presenting structures of NH_4_VO_3_ obtained from V_2_O_5_ in formamide using ammonium formate (a), ammonium acetate (b), and equimolar mixture of ammonium formate and ammonium acetate (c and d), Figure S6: SEM images presenting structures of NH_4_VO_3_ obtained in the reaction of V_2_O_5_ and ammonium formate carried out in the 1:1 (*v*/*v*) mixture of water and formamide, Figure S7: SEM image of commercial NH_4_VO_3_ used in a hydrothermal synthesis.
