# Supplementary material for: Tailoring the Size and Shape—New Path for Ammonium Metavanadate Synthesis
Source: Materials (Basel). 2019 Oct 21;12(20):3446. doi: 10.3390/ma12203446 (PMC6829540; doi:10.3390/ma12203446)
Supplement: Supplementary file 1 [file materials-12-03446-s001.pdf]

materials

Article

Tailoring the Size and Shape—New Path for Ammonium Metavanadate Synthesis

Marta Prześniak-Welenc *, Małgorzata Nadolska, Barbara Kościelska and Kamila Sadowska

Faculty of Applied Physics and Mathematics, Gdansk University of Technology, Narutowicza 11/12, 80-233 Gdansk, Poland; malgorzata.nadolska@pg.edu.pl (M.N.); barkosci@pg.edu.pl (B.K.); kamsadow@pg.edu.pl (K.S.)

* Correspondence: marta.welenc@pg.edu.pl; Tel: +48 583486606

Received: 23 September 2019; Accepted: 17 October 2019; Published: 21 October 2019

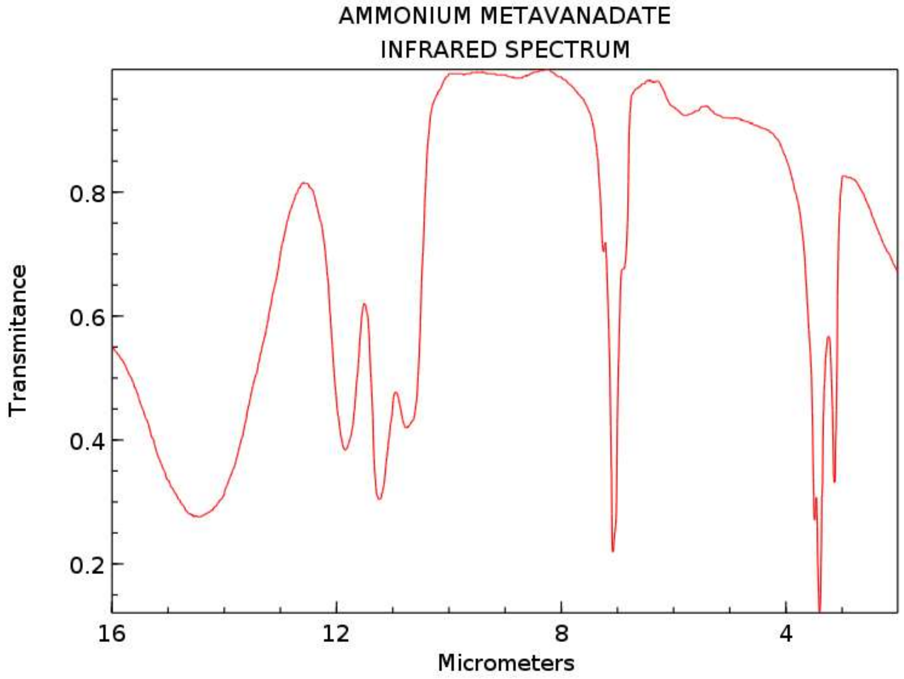

Figure S1. FTIR spectrum of ammonium metavanadate [1].

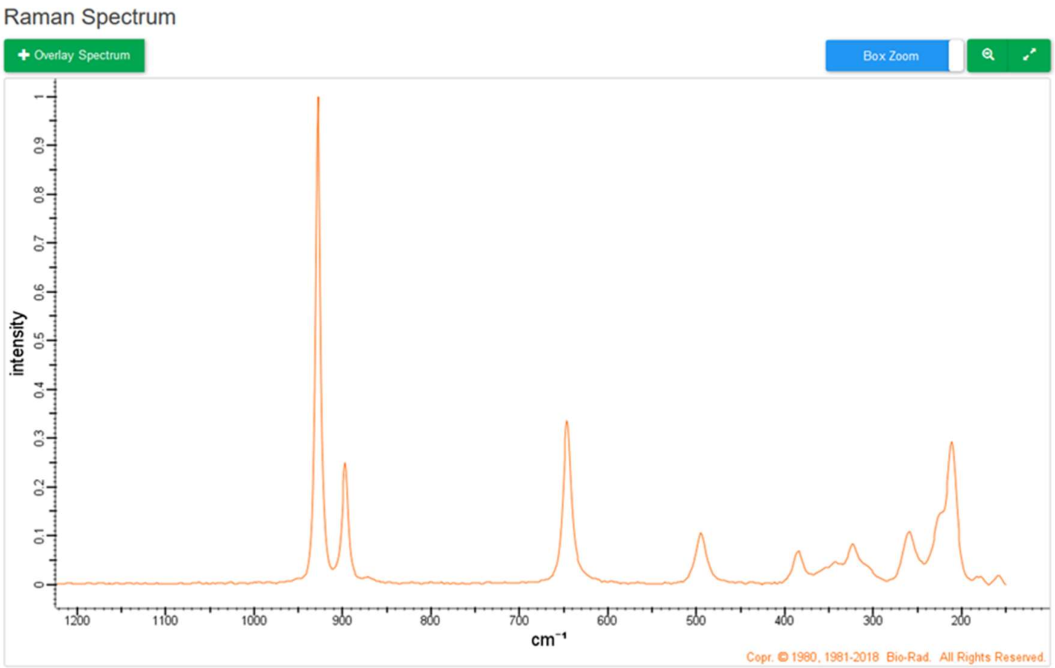

Figure S2. Raman spectrum of ammonium metavanadate.

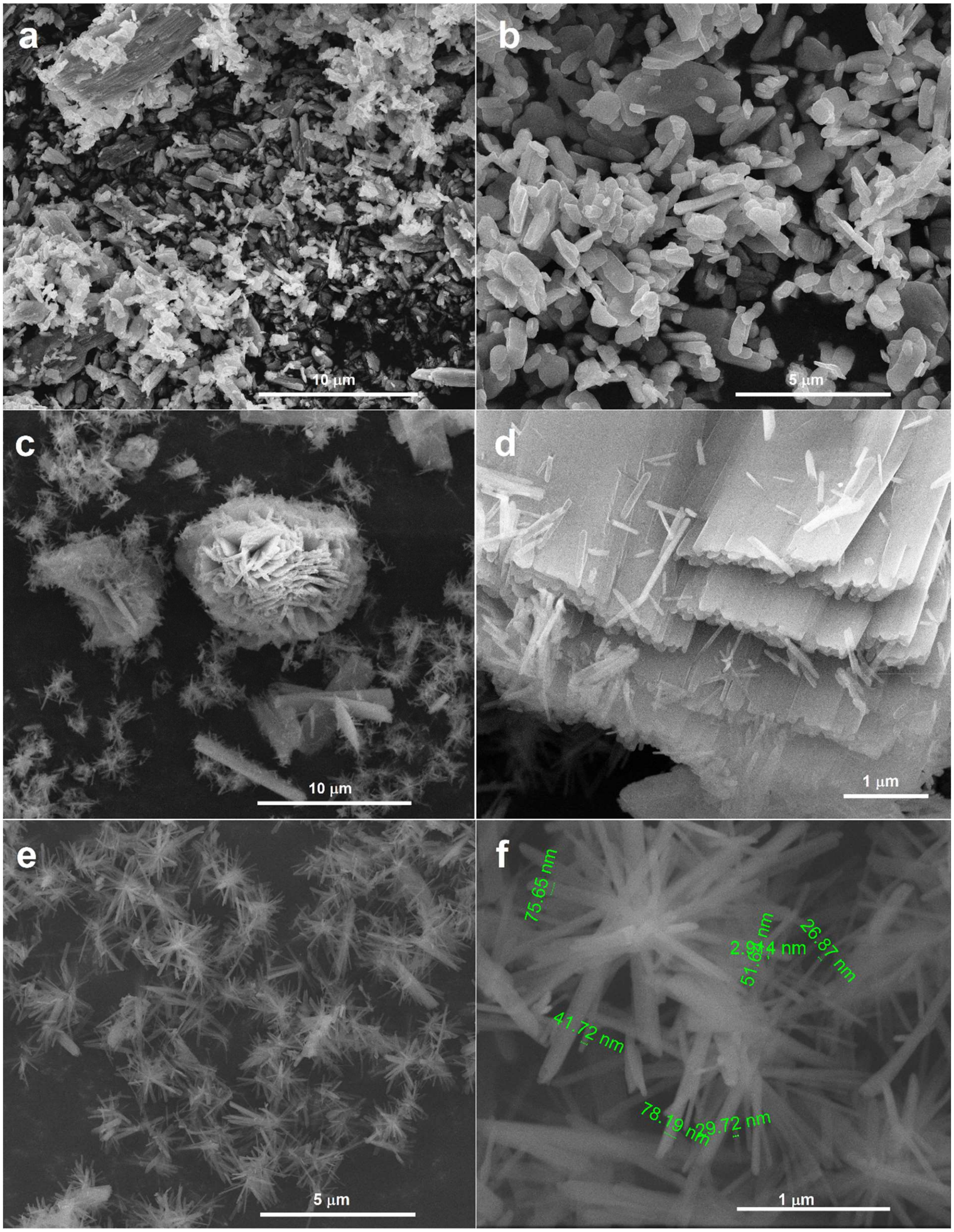

Figure S3. SEM images presenting the structure of V_2O_5 used as a precursor (**a**,**b**) and intermediates formed in the reaction of V_2O_5 with ammonium formate in formamide (**c**,**d**), leading to flower-like nanostructural crystals of NH_4VO_3 (**e**,**f**).

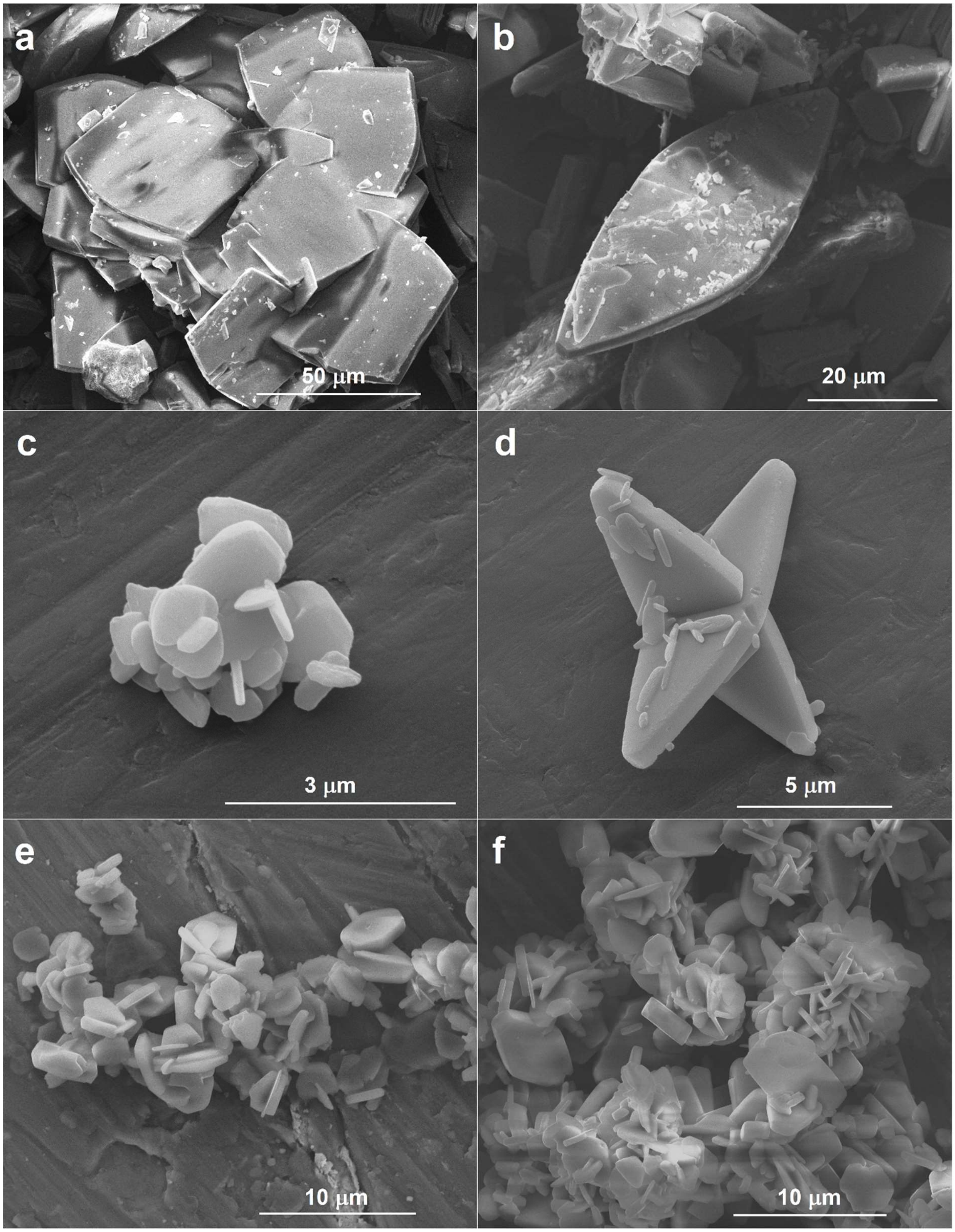

Figure S4. SEM images presenting structures of NH_4VO_3 obtained from V_2O_5 in water using ammonium formate (**a**,**b**), ammonium acetate (**c**,**d**), and equimolar mixture of ammonium formate and ammonium acetate (**e**,**f**).

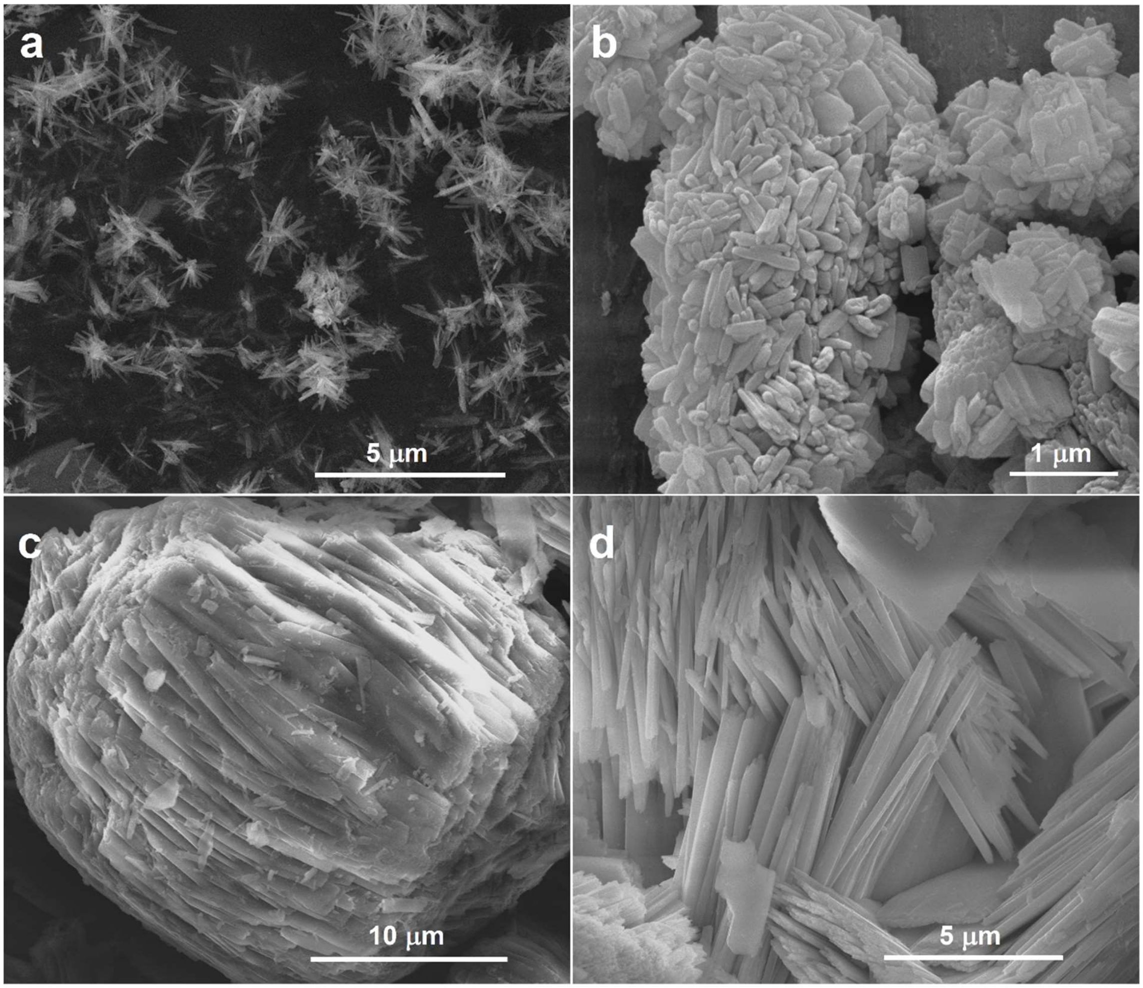

Figure S5. SEM images presenting structures of NH_4VO_3 obtained from V_2O_5 in formamide using ammonium formate (**a**), ammonium acetate (**b**), and equimolar mixture of ammonium formate and ammonium acetate (**c**,**d**).

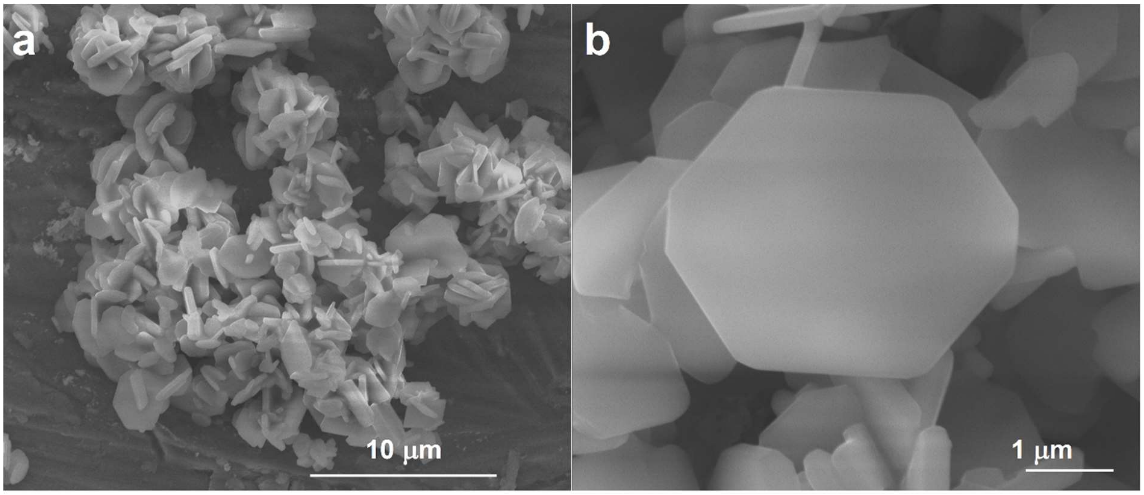

Figure S6. SEM images presenting structures of NH_4VO_3 obtained in the reaction of V_2O_5 and ammonium formate carried out in the 1:1 (*v*/*v*) mixture of water and formamide.

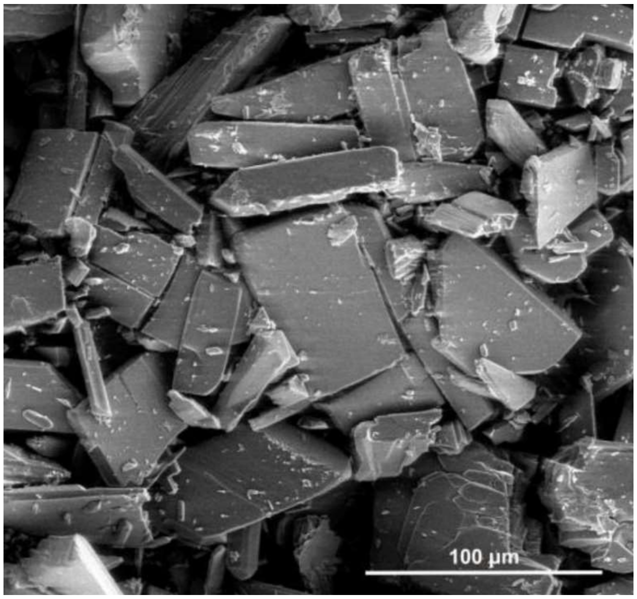

Figure S7. SEM image of commercial NH_4VO_3 used in a hydrothermal synthesis.

References:

1. NIST Chemistry WebBook; Available online: https://webbook.nist.gov/chemistry (accessed on 27 July 2019).

© 2019 by the authors. Submitted for possible open access publication under the terms and conditions of the Creative Commons Attribution (CC BY) license (http://creativecommons.org/licenses/by/4.0/).
